# Developing a framework for evidence-based grading and assessment of predictive tools for clinical decision support

**DOI:** 10.1186/s12911-019-0940-7

**Published:** 2019-10-29

**Authors:** Mohamed Khalifa, Farah Magrabi, Blanca Gallego

**Affiliations:** 1 Australian Institute of Health Innovation, Faculty of Medicine and Health Sciences, Macquarie University, Sydney, Australia; 2 Centre for Big Data Research in Health, Faculty of Medicine, Univerisity of New South Wales, Sydney, Australia

**Keywords:** Predictive analytics, Clinical prediction, Clinical decision support, Evidence-based medicine

## Abstract

**Background:**

Clinical predictive tools quantify contributions of relevant patient characteristics to derive likelihood of diseases or predict clinical outcomes. When selecting predictive tools for implementation at clinical practice or for recommendation in clinical guidelines, clinicians are challenged with an overwhelming and ever-growing number of tools, most of which have never been implemented or assessed for comparative effectiveness. To overcome this challenge, we have developed a conceptual framework to Grade and Assess Predictive tools (GRASP) that can provide clinicians with a standardised, evidence-based system to support their search for and selection of efficient tools.

**Methods:**

A focused review of the literature was conducted to extract criteria along which tools should be evaluated. An initial framework was designed and applied to assess and grade five tools: LACE Index, Centor Score, Well’s Criteria, Modified Early Warning Score, and Ottawa knee rule. After peer review, by six expert clinicians and healthcare researchers, the framework and the grading of the tools were updated.

**Results:**

GRASP framework grades predictive tools based on published evidence across three dimensions: 1) Phase of evaluation; 2) Level of evidence; and 3) Direction of evidence. The final grade of a tool is based on the highest phase of evaluation, supported by the highest level of positive evidence, or mixed evidence that supports a positive conclusion. Ottawa knee rule had the highest grade since it has demonstrated positive post-implementation impact on healthcare. LACE Index had the lowest grade, having demonstrated only pre-implementation positive predictive performance.

**Conclusion:**

GRASP framework builds on widely accepted concepts to provide standardised assessment and evidence-based grading of predictive tools. Unlike other methods, GRASP is based on the critical appraisal of published evidence reporting the tools’ predictive performance before implementation, potential effect and usability during implementation, and their post-implementation impact. Implementing the GRASP framework as an online platform can enable clinicians and guideline developers to access standardised and structured reported evidence of existing predictive tools. However, keeping GRASP reports up-to-date would require updating tools’ assessments and grades when new evidence becomes available, which can only be done efficiently by employing semi-automated methods for searching and processing the incoming information.

## Background

Modern healthcare is building upon information technology to improve the effectiveness, efficiency, and safety of healthcare and clinical processes [1–7]. In particular, clinical decision support (CDS) systems operate in three levels [8, 9]: 1) Managing information, through facilitating the reach to clinical knowledge, the search for, and the retrieval of relevant information needed to make clinical decisions, 2) Focusing users’ attention, through flagging abnormal results, providing lists of possible explanations for such results, or generating clinical and drug interaction alerts, and 3) Recommending specific actions and decisions, tailored for the clinical condition of the patient, which is often supported by clinical models and smart clinical guidelines.

Clinical predictive tools (here referred to simply as predictive tools) belong to the third level of CDS and include various applications ranging from the simplest manually applied clinical prediction rules to the most sophisticated machine learning algorithms [10, 11]. Through the processing of relevant clinical variables, clinical predictive tools derive the likelihood of diseases and predict their possible outcomes in order to provide patient specific diagnostic, prognostic, or therapeutic decision support [12, 13].

### Why do we need grading and assessment of predictive tools?

Traditionally, the selection of predictive tools for implementation in clinical practice has been conducted based on subjective evaluation of and exposure to particular tools [14, 15]. This is not optimal since many clinicians lack the required time and knowledge to evaluate predictive tools, especially as their number and complexity have increased tremendously in recent years.

More recently, there has been an increase in the mention and recommendation of selected predictive algorithms in clinical guidelines. However, this represents only a small proportion of the amount and variety of proposed clinical predictive tools, which have been designed for various clinical contexts, target many different patient populations and comprise a wide range of clinical inputs and techniques [16–18]. Unlike in the case of treatments, clinicians generating guidleines have no available methods to objectively summarise or interpret the evidence behind clinical predictive tools. This is made worse by the complex nature of the evaluation process itself and the variability in the quality of the published evidence [19–22].

Although most reported tools have been internally validated, only some have been externally validated and very few have been implemented and studied for their post-implementation impact on healthcare [23, 24]. Various studies and reports indicate that there is an unfortunate practice of developing new tools instead of externally validating or updating existing ones [13, 25–28]. More importantly, while a few pre-implementation studies compare similar predictive tools along some predictive performance measures, comparative studies for post-implementation impact or cost-effectiveness are very rare [29–38]. As a result, there is lack of a reference against which predictive tools can be compared or benchmarked [12, 39, 40].

In addition, decision makers need to consider the usability of a tool, which depends on the specific healthcare and IT settings in which it is embedded, and on users’ priorities and perspectives [41]. The usability of tools is consistently improved when the outputs are actionable or directive [42–45]. Moreover, clinicians are keen to know if a tool has been endorsed by certain professional organisations they follow, or recommended by specific clinical guidelines they know [26].

### Current methods for appraising predictive tools

Several methods have been proposed to evaluate predictive tools [13, 24, 46–60]. However, most of these methods are not based on the critical appraisal of the existing evidence. Two exceptions are the TRIPOD statement [61, 62], which provides a set of recommendations for the reporting of studies developing, validating, or updating predictive tools; and the CHARMS checklist [63], which provides guidance on critical appraisal and data extraction for systematic reviews of predictive tools. Both of these methods examine only the pre-implementation predictive performance of the tools, ignoring their usability and post-implementation impact. In addition, none of the currently available methods provide a grading system to allow for benchmarking and comparative effectiveness of tools.

On the other hand, looking beyond predictive tools, the GRADE framework, grades the quality of published scientific evidence and strength of clinical recommendations, in terms of their post-implementation impact. GRADE has gained a growing consensus, as an objective and consistent method to support the development and evaluation of clinical guidelines, and has increasingly been adopted worldwide [64, 65]. According to GRADE, information based on randomised controlled trials (RCTs) is considered the highest level of evidence. However, the level of evidence could be downgraded due to study limitations, inconsistency of results, indirectness of evidence, imprecision, or reporting bias [65–67]. The strength of a recommendation indicates the extent to which one can be confident that adherence to the recommendation will do more good than harm, it also requires a balance between simplicity and clarity [64, 68].

The aim of this study is to develop a conceptual framework for evidence-based grading and assessment of predictive tools. This framework is based on the critical appraisal of information provided in the published evidence reporting the evaluation of predictive tools. The framework should provide clinicians with standardised objective information on predictive tools to support their search for and selection of effective tools for their intended tasks. It should support clinicians’ informed decision making, whether they are implementing predictive tools at their clinical practices or recommending such tools in clinical practice guidelines to be used by other clinicians.

## Methods

Guided by the work of Friedman and Wyatt, and their suggested three phases approach, which became an internationally acknowledged standard for evaluating health informatics technologies [20, 21, 41, 69], we aimed to extract the main criteria along which predictive tools can be similarly evaluated before, during and after their implementation.

We started with a focused review of the literature in order to examine and collect the evaluation criteria, and measures proposed for the appraisal of predictive tools along these three phases. The concepts used in the search included “clinical prediction”, “tools”, “rules”, “models”, “algorithms”, “evaluation”, and “methods”. The search was conducted using four databases; MEDLINE, EMBASE, CINAHL and Google Scholar, with no specific timeframe. This literature review was then extended to include studies describing methods evaluating CDS systems and more generally, health information systems and technology. Following the general concepts of the PRISMA guidelines [70], the duplicates of the retrieved studies, from the four databases, were first removed. Studies were then screened, based on their titles and abstracts, for relevance, then the full text articles were assessed for eligibility and only the eligible studies were included in the review. We included three types of studies evaluating predictive tools and other CDS systems; 1) studies describing the methods or processes of the evaluation, 2) studies describing the phases of the evaluation, and 3) studies describing the criteria and measures used in the evaluation. The first author manually extracted the methods of evaluation described in each type of study, and this was then revised and confirmed by the second and last authors. Additional file 1: Figure S1 shows the process of study selection for inclusion in the focused review of the literature.

Using the extracted information, we designed an initial version of the framework and applied it to asses and grade five predictive tools. We reviewed the complete list of 426 tools published by the MDCalc medical reference website for decision support tools and applications and calculators (https://www.mdcalc.com) [71]. We excluded tools which are not clinical - their output is not related to providing individual patient care, such as scores of ED crowding and calculators of waiting times. We also excluded tools which are not predictive - their output is not the result of statistically generating new information but rather the result of a deterministic equation, such as calculators deriving a number from laboratory results. The five example tools were then randomly selected, using a random number generator [72], from a shorter list of 107 eligible predictive tools, after being alphabetically sorted and numbered.

A comprehensive and systematic search for the published evidence, on each of the five predictive tools, was conducted, using MEDLINE, EMBASE, CINAHL and Google Scholar, and refined in four steps. 1) The primary studies, describing the development of the tools, were first identified and retrieved. 2) All secondary studies that cited the primary studies or that referred to the tools’ names or to any of their authors, anywhere in the text, were retrieved. 3) All tertiary studies that cited the secondary studies or that were used as references by the secondary studies were retrieved. 4) Secondary and tertiary studies were examined to exclude non-relevant studies or those not reporting the validation, implementation or evaluation of the tools. After the four steps, eligible evidence was examined and grades were assigned to the predictive tools. Basic information about the tool, such as year of publication, intended use, target population, target outcome, and source and type of input data were extracted, from the primary studies, to inform the first “Tool Information” section of the framework. Eligible studies were then examined in detail for the reported evaluations of the predictive tools. Additional file 1: Figure S2 shows the process of searching the literature for the published evidence on the predictive tools.

**Fig. 1:**
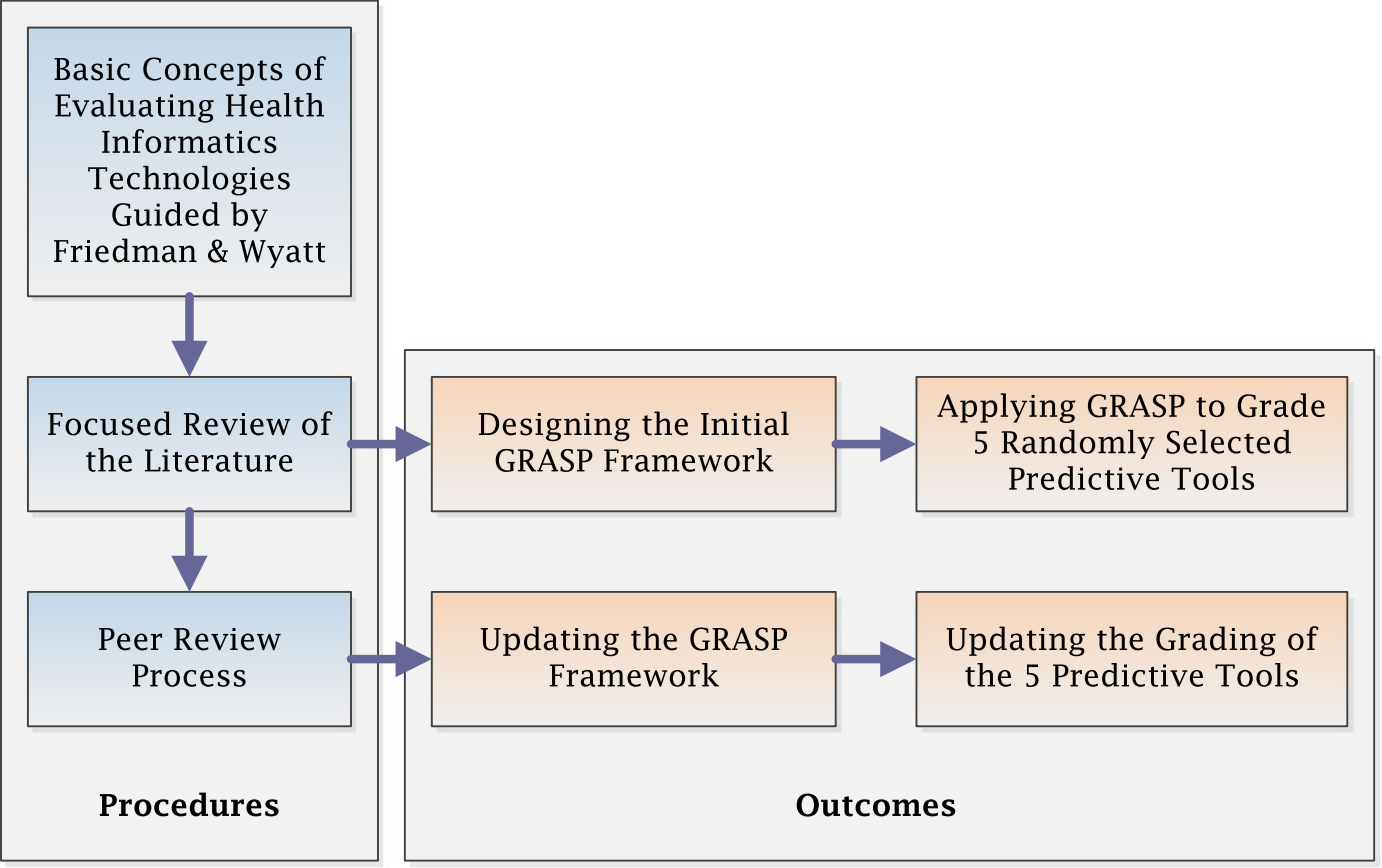
The GRASP Framework Development Flowchart

The framework and its application to the selected five predictive tools were then peer reviewed by six expert healthcare professionals. Three of these professionals are clinicians, who work in hospitals and have over 10 years of experience using CDS systems, while the other three are healthcare researchers, who work in research organisations and have over 20 years of experience in developing, implementing or evaluating CDS systems. The reviewers were provided with the framework’s concept design and its detailed report template. They were also provided with the summarised and detailed grading of the five predictive tools, as well as the justification and published evidence underpinning the grade assignment. After a brief orientation session, reviewers were asked to feedback on how much they agreed with each of the framework’s dimensions and corresponding evaluation criteria, the ‘Tool information’ section as well as the grading of the five exemplar predictive tools. The framework was then refined and the grading of the five predictive tools was updated based on the reviewers’ feedback. Figure 1 shows the flowchart of the GRASP framework overall development process.

## Results

### The focused review of literature

The search in the four databases, after removing the duplicates, identified a total of 831 studies. After screening the titles and abstracts, 647 studies were found not relevant to the topic. The full text of the remaining 184 studies were then examined to exclude non-eligible studies, which were 134 studies, based on the inclusion criteria. Only 50 studies were identified as eligible. Twenty three of the 50 studies described methods for the evaluation of predictive tools [13, 17, 24, 40, 44, 46–61, 63, 73], ten studies described the evaluation of CDS systems [2, 74–82], and 11 studies described the evaluation of hospital information systems and technology [83–93]. One study described the NASSS framework, a guideline to help predict and evaluate the success of healthcare technologies [94]; and five studies described the GRADE framework for evaluating clinical guidelines and protocols [64–68]. The following three subsections describe the methods used to evaluate predictive tools as described in the focussed literature review. A summary of the evaluation criteria and examples of corresponding measures for each phase can be found in Additional file 1: Table S1.

### Before implementation – predictive performance

During the development phase, the internal validation of the predictive performance of a tool is the first step to make sure that the tool is doing what it is intended to do [54, 55]. Predictive performance is defined as the ability of the tool to utilise clinical and other relevant patient variables to produce an outcome that can be used to supports diagnostic, prognostic or therapeutic decisions made by clinicians and other healthcare professionals [12, 13]. The predictive performance of a tool is evaluated using measures of discrimination and calibration [53]. Discrimination refers to the ability of the tool to distinguish between patients with and without the outcome under consideration. This can be quantified with measures such as sensitivity, specificity, and the area under the receiver operating characteristic curve – AUC (or concordance statistic, c). The D-statistic is a measure of discrimination for time-to-event outcomes, which is commonly used in validating the predictive performance of prognostic models using survival data [95]. The log-rank test, or sometimes referred to as the Mantel-Cox test, is used to establish if the survival distributions of two samples of patients are statistically different. They are commonly used to validate the discrimination power of clinical prognostic models [96]. On the other hand, calibration refers to the accuracy of prediction, and indicates the extent to which expected and observed outcomes agree [48, 56]. Calibration is measured by plotting the observed outcome rates against their corresponding predicted probabilities. This is usually presented graphically with a calibration plot that shows a calibration line, which can be described with a slope and an intercept [97]. It is sometimes summarised using the Hosmer-Lemeshow test or the Brier score [98]. To avoid over-fitting, tools’ predictive performance must always be assessed out-of-sample, either via cross-validation or bootstrapping [56]. Of more interest than the internal validity is the external validity (reliability or generalisability), where the predictive performance of a tool is estimated in independent validation samples of patients from different populations [52].

### During implementation – potential effect & usability

Before wide implementation, it is important to learn about the estimated potential effect of a predictive tool, when used in the clinical practice, on three main categories of measures: 1) Clinical effectiveness, such as improving patient outcomes, estimated through clinical effectiveness studies, 2) healthcare efficiency, including saving costs and resources, estimated through feasibility and cost-effectiveness studies, and 3) patient safety, including minimising complications, side effects, and medical errors. These categories are defined by the Institute of Medicine as objectives for improving healthcare performance and outcomes, and are differently prioritised by clinicians, healthcare professionals and health administrators [99, 100]. The potential effect, of a predictive tool, is defined as the expected, estimated or calculated impact of using the tool on different healthcare aspects, processes or outcomes, assuming the tool has been successfully implemented and is used in the clinical practice, as designed by its developers [41, 101]. A few predictive tools have been studied for their potential to enhance clinical effectiveness and improve patient outcomes. For example, the spinal manipulation clinical prediction rule was tested, before implementation, on a small sample of patients to identify those with low back pain most likely to benefit from spinal manipulation [102]. Other tools have been studied for their potential to improve healthcare efficiency and save costs. For example, using a decision analysis model, and assuming all eligible children with minor blunt head trauma were managed using the CHALICE rule (Children’s Head Injury Algorithm for the Prediction of Important Clinical Events), it was estimated that CHALICE would reduce unnecessary expensive head computed tomography (CT) scans, by 20%, without risking patients’ health [103–105]. Similarly, the use of the PECARN (Paediatric Emergency Care Applied Research Network) head injury rule was estimated to potentially improve patient safety through minimising the exposure of children to ionising radiation resulting in fewer radiation-induced cancers and lower net quality adjusted life years loss [106, 107].

In addition, it is important to learn about the usability of predictive tools. Usability is defined as the extent to which a system can be used by the specified users to achieve specified and quantifiable objectives in a specified context of use [108, 109]. There are several methods to make a system more usable and many definitions have been developed, based on the perspective of what usability is and how it can be evaluated, such as the mental effort needed and the user attitude or the user interaction, represented in the easiness of use and acceptability of systems [110, 111]. Usability can be evaluated through measuring the effectiveness of task management with accuracy and completeness, measuring efficiency of utilising resources in completing tasks and measuring users’ satisfaction, comfort with, and positive attitudes towards, the use of the tools [112, 113]. More advanced techniques, such as think aloud protocols and near live simulations, are recently used to evaluate usability [114]. Think aloud protocols are a major method in usability testing, since they produce a larger set of information and a richer content. They are conducted either retrospectively or concurrently, where each method has its own way of detecting usability problems [115]. The near live simulations provide users, during testing, with an opportunity to go through different clinical scenarios while the system captures interaction challenges and usability problems [116, 117]. Some researchers add learnability, memorability and freedom of errors to the measures of usability. Learnability is an important aspect of usability and a major concern in the design of complex systems. It is the capability of a system to enable the users to learn how to use it. Memorability, on the other hand, is the capability of a system to enable the users to remember how to use it, when they return back. Learnability and memorability are measured through subjective survey methods, asking users about their experience after using systems, and can also be measured by monitoring users’ competence and learning curves over successive sessions of system usage [118, 119].

### After implementation – post-implementation impact

Some predictive tools have been implemented and used in the clinical practice for years, such as the PECARN head injury rule or the Ottawa knee and ankle rules [120–122]. In such cases, clinicians might be interested to learn about their post-implementation impact. The post-implementation impact of predictive tools is defined as the achieved change or influence, of a predictive tool, on different healthcare aspects, processes or outcomes, after the tool has been successfully implemented and used in the clinical practice, as designed by its developers [2, 42]. Similar to the measures of potential effect, post-implementation impact is reported along three main categories of measures: 1) Clinical effectiveness, such as improving patient outcomes, 2) Healthcare efficiency, such as saving costs and resources, and 3) Patient safety, such as minimising complications, side effects, and medical errors. These three categories of post-implementation impact measures are differently prioritised by clinicians, healthcare professionals and health administrators. In this phase of evaluation, we follow the main concepts of the GRADE framework, where the level of evidence for a given outcome is firstly determined by the study design [64, 65, 68]. High quality experimental studies, such as randomised and nonrandomised controlled trials, and the systematic reviews of their findings, come on top of the evidence levels followed by observational well-designed cohort or case-control studies and lastly subjective studies, opinions of respected authorities, and reported of expert committees or panels [65–67]. For simplicity, we did not include GRADE’s detailed criteria for higher and lower quality of studies. However, effect sizes and potential biases are reported as part of the framework, so that consistency of findings, trade-offs between benefits and harms, and other considerations can also be assessed.

### Developing the GRASP framework

Our suggested GRASP framework (abbreviated from Grading and Assessment of Predictive Tools) is illustrated in Table 1. Based on published evidence, the GRASP framework uses three dimensions to grade predictive tools: ***Phase of Evaluation*,** ***Level of Evidence*** **and** ***Direction of Evidence***.

**Table 1:**
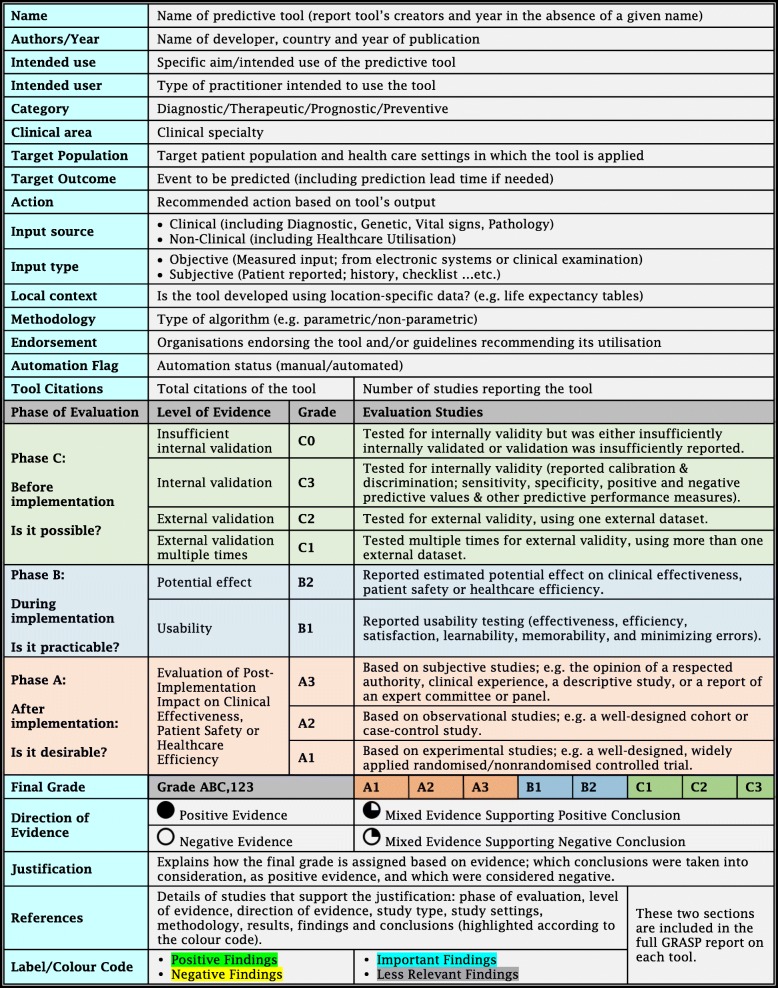
The GRASP Framework Detailed Report

#### Phase of evaluation

Assigns a letter A, B and/or C based on the highest phase of evaluation reported in the published literature. A tool is assigned the lowest phase, C, if its predictive performance has been tested and reported for validity; phase B if its usability and/or potential impact have been validated; and the highest phase, A, if it has been implemented in clinical practice and its post-implementation impact has been reported.

#### Level of evidence

Assigns a numerical score within each phase of evaluation based on the level of evidence associated with the evaluation process. Tools in phase C of evaluation can be assigned three levels. A tool is assigned the lowest level, C3, if it has been tested for internal validity; C2 if it has been tested for external validity once; and C1 if it has been tested for external validity multiple times. Similarly, tools in phase A of evaluation can be assigned the lowest level of evidence, A3, if their post-implementation impact has been evaluated only through subjective or descriptive studies; A2 if it has been evaluated via observational studies; and A1 if post-implementation impact has been measured using experimental evidence. Tools in phase B of evaluation are assigned grade B2 if they have been tested for potential impact and B1 if they have been tested for usability. Effect sizes for each outcome of interest together with study type, clinical settings and patient populations are also reported.

#### Direction of evidence

Due to the large heterogeneity in study design, outcome measures and patient subpopulations contained in the studies, synthesising measures of predictive performance, usability, potential effect or post-implementation impact into one quantitative value is not possible. Furthermore, acceptable values of predictive performance or post-implementation impact measures depend on the clinical context and the task at hand. For example, tools like Ottawa knee rule [122] and Wells’ criteria [123, 124] are considered effective only when their sensitivity is very close to 100%, since their task is to identify patients with fractures or pulmonary embolism before sending them home. On the other hand, tools like LACE Index [125] and Centor score [126] are accepted to show sensitivities of around 70%, since their tasks, to predict 30 days readmission risk or identify that pharyngitis is bacterial, aim to screen patients who may benefit from further interventions. Therefore, for each phase and level of evidence, we assign a direction of evidence, based on the conclusions reported in the studies, and provide the user with the option to look at the summary of the findings for further information.

Positive evidence is assigned when all studies reported positive conclusions while negative evidence is assigned when all studies reported negative or equivocal conclusions. In the presence of mixed evidence, studies are farther ranked according to their quality as well as their degree of matching with the orginal tool specifications (namely target population, target outcome and settings). Mixed evidence is then classified considering this ranking as supporting an overall positive conclusion or supporting an overall negative conclusion. Details and illustration of this protocol can be found in Additional file 1: Table S2 and Figure S3.

**Fig. 2:**
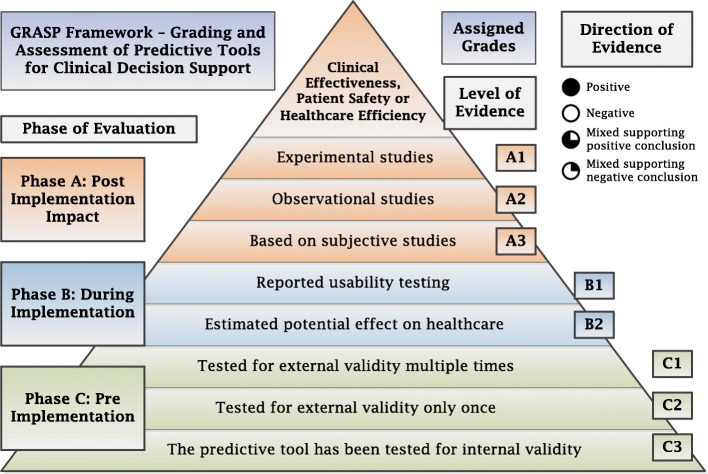
The GRASP Framework Concept

The final grade of a predictive tool is based on the highest phase of evaluation, supported by the highest level of positive evidence, or mixed evidence that supports an overall positive conclusion. Figure 2 shows the GRASP framework concept; a visual presentation of the framework three dimensions, phase of evaluation, level of evidence, and direction of evidence, explaining how each tool is assigned the final grade. Table 1 shows the GRASP framework detailed report.

### Applying the GRASP framework to grade five predictive tools

In order to show how GRASP works, we applied it to grade five randomly selected predictive tools; LACE Index for Readmission [125], Centor Score for Streptococcal Pharyngitis [126], Wells’ Criteria for Pulmonary Embolism [123, 124, 127], The Modified Early Warning Score (MEWS) for Clinical Deterioration [128] and Ottawa Knee Rule [122]. In addition to these seven primary studies, describing the development of the five predictive tools, our systematic search for the published evidence revealed a total of 56 studies; validating, implementing, and evaluating the five predictive tools. The LACE Index was evaluated and reported in six studies, the Centor Score in 14 studies, the Wells’ Criteria in ten studies, the MEWS in 12 studies, and the Ottawa Knee Rule in 14 studies. To apply the GRASP framework and assign a grade to each predictive tool, the following steps were conducted; 1) The primary study or studies were first examined for the basic information about the tool and the reported details of development and validation. 2) Other studies were examined for their phases of evaluation, levels of evidence and direction of evidence. 3) Mixed evidence was sorted into positive or negative. 4) The final grade was assigned and supported by the detailed justification. A summary of grading the five tools is shown in Table 2 and a detailed GRASP report on each tool is provided in the Additional file 1: Tables S3-S7.

**Table 2:**
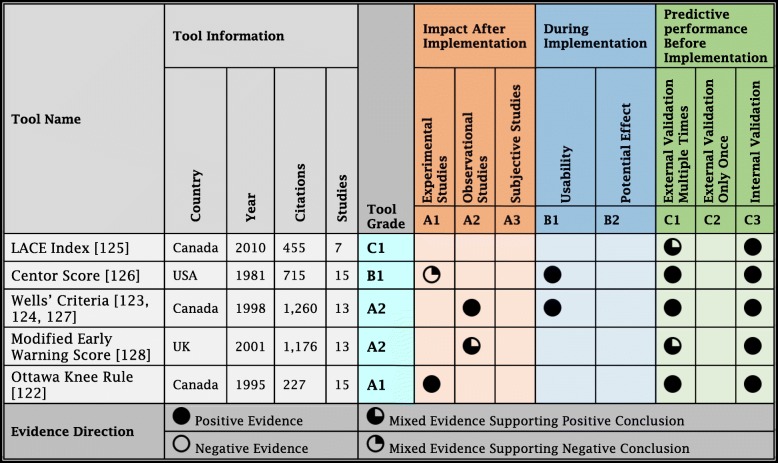
Summary of Grading the Five Predictive Tools

**LACE Index** is a prognostic tool designed to predict 30 days readmission or death of patients after discharge from hospitals. It uses multivariable logistic regression analysis of four administrative data elements; length of stay, admission acuity, comorbidity (Charlson Comorbidity Index) and emergency department (ED) visits in the last 6 months, to produce a risk score [125]. The tool has been tested for external validity twice; using a sample of 26,045 patients from six hospitals in Toronto and a sample of 59,652 patients from all hospitals in Alberta, Canada. In both studies, the LACE Index showed positive external validity and superior predictive performance to the previous similar tools endorsed by the Centres for Medicare and Medicaid Services in the United States [129, 130].

Two studies examined the predictive performance of LACE Index on small sub-population samples; 507 geriatric patients in the United Kingdom and 253 congestive heart failure patients in the United States, and found that the index performed poorly [131, 132]. Two more studies reported that the LACE Index performed well but not better that their own developed tools [133, 134]. Using the mixed evidence protocol, the mixed evidence here supports external validity, since the two negative conclusion studies have been conducted on very small samples of patients and on different subpopulations than the one the LACE Index was developed for. There was no published evidence on the usability, potential effect or post-implementation impact of the LACE Index. Accordingly, the LACE Index has been assigned Grade C1.

**Centor Score** is a diagnostic tool that uses a rule-based algorithm on clinical data to estimate the probability that pharyngitis is streptococcal in adults who present to the ED complaining of sore throat [126]. The score has been tested for external validity multiple times and all the studies reported positive conclusions [135–142]. This qualifies Centor score for Grade C1. One study conducted a multicentre cluster RCT usability testing of the integration of Centor score into electronic health records. The study used “Think Aloud” testing with ten primary care providers, post interaction surveys in addition to screen captures and audio recordings to evaluate usability. Within the same study, another “Near Live” testing, with eight primary care providers, was conducted. Conclusions reported positive usability of the tool and positive feedback of users on the easiness of use and usefulness [143]. This qualifies Centor score for Grade B1.

Evidence of the post-implementation impact of Centor score is mixed. One RCT conducted in Canada reported a clinically important 22% reduction in overall antibiotic prescribing [144]. Four other studies, three of which were RCTs, reported that implementing Centor score did not reduce antibiotic prescribing in clinical practice [145–148]. Using the mixed evidence protocol, we found that the mixed evidence does not support positive post-implementation impact of Centor score. Therefore, Centor score has been assigned Grade of B1.

**Wells’ Criteria** is a diagnostic tool used in the ED to estimate pre-test probability of pulmonary embolism [123, 124]. Using a rule-based algorithm on clinical data, the tool calculates a score that excludes pulmonary embolism without diagnostic imaging [127]. The tool was tested for external validity multiple times [149–153] and its predictive performance has been also compared to other predictive tools [154–156]. In all studies, Wells’ criteria was reported externally valid, which qualifies it for Grade C1. One study conducted usability testing for the integration of the tool into the electronic health record system of a tertiary care centre’s ED. The study identified a strong desire for the tool and received positive feedback on the usefulness of the tool itself. Subjects responded that they felt the tool was helpful, organized, and did not compromise clinical judgment [157]. This qualifies Wells’ criteria for Grade B1. The post-implementation impact of Well’s Criteria on efficiency of computed tomography pulmonary angiography (CTPA) utilisation has been evaluated through an observational before-and-after intervention study. It was found that the Well’s Criteria significantly increased the efficiency of CTPA utilisation and decreased the proportion of inappropriate scans [158]. Therefore, Well’s Criteria has been assigned Grade A2.

**The Modified Early Warning Score (MEWS)** is a prognostic tool for early detection of inpatients’ clinical deterioration and potential need for higher levels of care. The tool uses a rule-based algorithm on clinical data to calculate a risk score [128]. The MEWS has been tested for external validity multiple times in different clinical areas, settings and populations [159–165]. All studies reported that the tool is externally valid. However, one study reported MEWS poorly predicted the in-hospital mortality risk of patients with sepsis [166]. Using the mixed evidence protocol, the mixed evidence supports external validity, qualifying MEWS for Grade C1. No literature has been found regarding its usability or potential effect.

The MEWS has been implemented in different healthcare settings. One observational before-and-after intervention study failed to prove positive post-implementation impact of the MEWS on patient safety in acute medical admissions [167]. However, three more recent observational before-and-after intervention studies reported positive post-implementation impact of the MEWS on patient safety. One study reported significant increase in frequency of patient observation and decrease in serious adverse events after intensive care unit (ICU) discharge [168]. The second reported significant increase in frequency of vital signs recording, 24 h post-ICU discharge and 24 h preceding unplanned ICU admission [169]. The third, an 8 years study, reported that the post-implementation 4 years showed significant reductions in the incidence of cardiac arrests, the proportion of patients admitted to ICU and their in-hospital mortality [170]. Using the mixed evidence protocol, the mixed evidence supports positive post-implementation impact. The MEWS has been assigned Grade A2.

**Ottawa Knee Rule** is a diagnostic tool used to exclude the need for an X-ray for possible bone fracture in patients presenting to the ED, using a simple five items manual check list [122]. It is one of the oldest, most accepted and successfully used rules in CDS. The tool has been tested for external validity multiple times. One systematic review identified 11 studies, 6 of them involved 4249 adult patients and were appropriate for pooled analysis, showing high sensitivity and specificity predictive performance [171]. Furthermore, two studies discussed the post-implementation impact of Ottawa knee rule on healthcare efficiency. One nonrandomised controlled trial with before-after and concurrent controls included a total of 3907 patients seen during two 12-month periods before and after the intervention. The study reported that the rule decreased the use of knee radiography without patient dissatisfaction or missed fractures and was associated with reduced waiting times and costs per patient [172]. Another nonrandomised controlled trial reported that the proportion of ED patients referred for knee radiography was reduced. The study also reported that the practice based on the rule was associated with significant cost savings [173]. Accordingly, the Ottawa knee rule has been assigned Grade A1.

In the Additional file 1, a summary of the predictive performance of the five tools is shown in Additional file 1: Table S8. The c-statistics of LACE Index, Centor Score, Wells’ Criteria and MEWS are reported in Additional file 1: Figure S4. The usability of Centor Score and Wells Criteria are reported in Additional file 1: Table S9 and post-implementation impact of Wells Criteria, MEWS and Ottawa knee rule is reported in Additional file 1: Table S10.

### Peer review of the GRASP framework

On peer-review, experts found the GRASP framework logical, helpful and easy to use. The reviewers strongly agreed to all criteria used for evaluation. The reviewers suggested adding more specific information about each tool, such as the author’s name, the intended user of the tool and the recommended action based on the tool’s findings. The reviewers showed a clear demand for knowledge regarding the applicability of tools to their local context. Two aspects were identified and discussed with the reviewers. Firstly, the operational aspect of how easy it would be to implement a particular tool and if the data required to use the tool is readily available in their clinical setting. Secondly, the validation aspect of adopting a tool developed using local predictors, such as life expectancy (which is location specific) or information based on billing codes (which is hospital specific). Following this discussion, elements related to data sources and context were added to the information section of the framework. One reviewer suggested assigning grade C0 to the reported predictive tools that did not meet C3 criteria, i.e. those tools which were tested for internally validity but were either insufficiently internally validated or the internal validation was insufficiently reported in the study, in order to differentiate them from those tools for which neither predictive performance nor post-implementation impact have been reported in the literature.

## Discussion

### Brief summary

It is challenging for clinicians to critically evaluate the growing number of predictive tools proposed to them by colleagues, administrators and commercial entities. Although most of these tools have been assessed for predictive performance, only a few have been implemented or evaluated for comparative predictive performance or post-implementation impact. In this manuscript, we present GRASP, a framework that provides clinicians with an objective, evidence-based, standardised method to use in their search for, and selection of tools. GRASP builds on widely accepted concepts, such as Friedman and Wyatt’s evaluation approach [20, 21, 41, 69] and the GRADE system [64–68].

The GRASP framework is composed of two parts; 1) the GRASP framework concept, which shows the grades assigned to predictive tools based on the three dimensions: Phase of Evaluation, Level of Evidence and Direction of Evidence, and 2) the GRASP framework detailed report, which shows the detailed quantitative information on each predictive tool and justifies how the grade was assigned. The GRASP framework is designed for two levels of users: 1) Expert users, who will use the framework to assign grades to predictive tools and report their details, through the critical appraisal of published evidence about these tools. This step is essential to provide decision making clinicians with grades and detailed reports on predictive tools. Expert users include healthcare researchers who specialise in evidence-based methods and have experience in developing, implementing or evaluating predictive tools. 2) End users, who will use the GRASP framework detailed report of tools and their final grades, produced by expert users, to compare existing predictive tools and select the most suitable tool(s) for their predictive tasks, target objectives, and desired outcomes. End users include clinicians and other healthcare professionals involved in the decision making and selection of predictive tools for implementation at their clinical practice or for recommendation in clinical practice guidelines to be used by other clinicians and healthcare professionals. The two described processes; the grading of predictive tools by expert users and the selection decisions made by end users, should occur before the recommended tools are made available for use in the hands of the practicing clinicians.

### Comparison with previous literature

Previous approaches to the appraisal of predictive tools from the published literature, namely the TRIPOD statement [61, 62] and the CHARMS checklist [63], examine only their predictive performance, ignoring their usability and post-implementation impact. On the other hand, the GRADE framework appraises the published literature in order to evaluate clinical recommendations based on their post-implementation impact [64–68]. More broadly, methods for the evaluation of health information systems and technology focus on the integration of systems into tasks, workflows and organisations [84, 91]. The GRASP framework takes into account all phases of the development and translation of a predictive algorithm: predictive performance before implementation, using similar concepts as those utilised in TRIPOD and CHARMS; usability and potential effect during implementation, and post-implementation impact on patient outcomes and processes of care after implementation, using similar concepts as those utilised in the GRADE system. The GRASP grade is not the result of combining and synthesising selected measures of predictive performance (e.g. AUC), potential effect (e.g. potential saved money), usability (e.g. user satisfaction) or post-implementation impact (e.g. increased efficacy) from the existing literature, like in a meta-analysis; but rather the result of combining and synthesising the reported qualitative conclusions.

Walker and Habboushe, at the MDCalc website; classified and reported the most commonly used medical calculators and other clinical decision support applications. However, the website does not provide users with a structured grading system or an evidence-based method for the assessment of the presented tools. Therefore, we believe that our proposed framework can be adopted and used by MDCalc, and similar clinical decision support resources, to grade their tools.

### Quality of evidence and conflicting conclusions

One of the main challenges in assessing and comparing clinical predictive tools is dealing with the large variability in the quality and type of studies in the published literature. This heterogeneity makes it impractical to quantitatively synthesise measures of predictive performance, usability, potential effect or post-implementation impact into single numbers. Furthermore, as discussed earlier, a particular value of a predictive performance metric that is considered good for some tasks and clinical settings may be considered insufficient for others. In order to avoid complex decisions regarding the quality and strength of reported measures, we chose to assign a direction of evidence, based on positive or negative conclusions as reported in the studies under consideration, since synthesizing qualitative conclusions is the only available option which adds some value. We then provide the end user with the option to look at a summary of the reported measures, in the GRASP detailed report, for further details.

It is not uncommon to encounter conflicting conclusions when a tool has been validated in different patient subpopulations. For example, LACE Index for readmission showed positive external validity when tested in adult medical inpatients [129, 130], but showed poor predictive performance when tested in a geriatric subpopulation [131]. Similarly, the MEWS for clinical deterioration demonstrated positive external validity when tested in emergency patients [159, 161, 165], medical inpatients [160, 164], surgical inpatients [162], and trauma patients [163], but not when tested in a subpopulation of patients with acute sepsis [166]. Part of these disagreements could be explained by changes in the distributions of important predictors, which affect the discriminatory power of the algorithms. For example, sepsis patients have similarly disturbed physiological measures such as those used to generate MEWS. In addition, conflicting conclusions may be encountered when a study examines the validity of a proposed tool in a healthcare setting or outcome different from those the tool was primarily developed for.

### Integration and socio-technical context

As is the case with other healthcare interventions, examining the post-implementation impact of predictive tools is challenging, since it is confounded by co-occurrent socio-technical factors [174–176]. This is complicated further by the fact that predictive tools are often integrated into electronic health record systems, since this facilitates their use, and are influenced by their usability [42, 177]. The usability, therefore, is an essential and major contributing factor in the wide acceptance and successful implementation of predictive tools and other CDS systems [42, 178]. It is clearly essential to involve user clinicians in the design and usability evaluations of predictive tools before their implementation. This should eliminate their concerns that integrating predictive tools into their workflow would increase their workload, consultation times, or decrease their efficiency and productivity [179].

Likewise, well designed post-implementation impact evaluation studies are required in order to explore the influence of organisational factors and local differences on the success or failure of predictive tools [180, 181]. Data availability, IT systems capabilities, and other human knowledge and organisational regulatory factors are crucial for the adoption, acceptance, and successful implementation of predictive tools. These factors and differences need to be included in the tools assessments, as they are important when making decisions about selecting predictive tools, in order to estimate the feasibility and resources needed to implement the tools. We have to acknowledge that it is not possible to include such wide range of variables in deciding or presenting the grades assigned by the framework to the predictive tools, which remain simply at a high-level. However, all the necessary information, technical specifications, and requirements of the tools, as reported in the published evidence, should be fully accessible to the users, through the framework's detailed reports on the predictive tools. Users can compare such information, of one or more tools, to what they have at their healthcare settings, then make selection and implementation decisions.

### Local data

There is a challenging trade-off between the generalisability and the customisation of a given predictive tool. Some algorithms are developed using local data. For example, Bouvy’s prognostic model, for mortality risk in patients with heart failure, uses life quality and expectancy scores from the Netherlands [182]. Similarly, Fine’s prediction rule identifies low-risk patients with community-acquired pneumonia based on national rates of acquired infections in the United States [183]. This necessitates adjustment of the algorithm to the local context, therefore producing a new version of the tool, which requires re-evaluation.

### Other considerations

GRASP evaluates predictive tools based on the critical appraisal of the existing published evidence. Therefore, it is subject to publication bias, since statistically positive results are more likely to be published than negative or null results [184, 185]. The usability and potential effect of predictive tools are less studied and hence the published evidence needed to support level B of the grading system is often lacking. We have nevertheless chosen to keep this element in GRASP since it is an essential part of the safety evaluation of any healthcare technology. It also allows for early redesign and better workflow integration, which leads to higher utilisation rates [114, 157, 186]. By keeping it, we hope to encourage tool developers and evaluators to increase their execution and reporting of these type of studies.

The grade assigned to a tool provides relevant evidence-based information to guide the selection of predictive tools for clinical decision support, but it is not prescriptive. An A1 tool is not always better than an A2 tool. A user may prefer an A2 tool showing improved patient safety in two observational studies rather than an A1 tool showing reduced cost in one experimental study. The grade is a code (not an ordinal quantity) that provides information on three relevant dimensions: phase of evaluation, level of evidence, and direction of evidence as reported in the literature.

### Study limitations and future work

One of the limitations of the GRASP framework is that the Direction of Evidence dimension is based on the conclusions of the considered studies on each predictive tool, which confers some subjectivity to this dimension. However, the end-user clinicians are provided with the full details of all available studies on each tool, through the GRASP detailed report, where they can access the required objective information to support their decisions. In addition, we applied the GRASP framework to only five predictive tools and consulted a small number of healthcare experts for their feedback. This could have limited the conclusions about the framework’s coverage and/or validity. Although GRASP framework is not a predictive tool, it could be thought of as a technology of Grade C3, since it has only been internally validated after development. However, conducting a large-scale validation study of the framework, extending the application of the framework to a larger number of predictive tools, and studying its effect on end-users’ decisions is out of the scope of this study and is left for future work.

To validate, update, and evaluate the GRASP framework, the authors are currently working on three more studies. The first study should validate the design and content of the framework, through seeking the feedback of a wider international group of healthcare experts, who have published work on developing, implementing or evaluating predictive tools. This study should help to update the criteria used, by the framework, to grade predictive tools and improve the details provided, by the framework, to the end users. The second study should evaluate the impact of using the framework on improving the decisions made by clinicians, regarding evaluating and selecting predictive tools. The experiment should compare the performance and outcomes of clinicians’ decisions with and without using the framework. Finally, the third study aims to apply the framework to a larger consistent group of predictive tools, used for the same clinical task. This study should show how the framework provides clinicians with an evidence-based method to compare, evaluate and select predictive tools, through reporting and grading tools based on the critical appraisal of published evidence.

## Conclusion

The GRASP framework builds on widely accepted concepts to provide standardised assessment and evidence-based grading of predictive tools. Unlike other methods, GRASP is based on the critical appraisal of published evidence reporting the tools’ predictive performance before implementation, potential effect and usability during implementation, and their post-implementation impact. Implementing the GRASP framework as an online platform can enable clinicians and guideline developers to access standardised and structured reported evidence of existing predictive tools. However, keeping the GRASP framework reports up-to-date would require updating tools’ assessments and grades when new evidence becomes available. Therefore, a sustainable GRASP system would require employing automated or semi-automated methods for searching and processing the incoming published evidence. Finally, we recommend that GRASP framework be applied to predictive tools by working groups of professional organisations, in order to provide consistent results and increase reliability and credibility for end users. These professional organisations should also be responsible for making their associates aware of the availability of such evidence-based information on predictive tools, in a similar way of announcing and disseminating clinical practice guidelines.

## Supplementary information

**Additional file 1: Table S1.** Phases, Criteria, and Measures of Evaluating Predictive Tools. **Table S2.** Evaluating Evidence Direction Based on the Conclusions of Studies. **Table S3.** LACE Index for Readmission – Grade C1. **Table S4.** Centor Score for Streptococcal Pharyngitis – Grade B1. **Table S5.** Wells’ Criteria for Pulmonary Embolism – Grade A2. **Table S6.** Modified Early Warning Score (MEWS) – Grade A2. **Table S7.** Ottawa Knee Rule – Grade A1. **Table S8.** Predictive Performance of the Five Tools – Before Implementation. **Table S9.** Usability of Two Predictive Tools – During Implementation. **Table S10.** Post-Implementation Impact of Three Predictive Tools. **Figure S1.** Study Selection for the Focused Review of Literature. **Figure S2.** Searching the Literature for Published Evidence on Predictive Tools. **Figure S3.** The Mixed Evidence Protocol. **Figure S4.** Reported C-Statistic of LACE Index, Centor Score, Wells Criteria and MEWS [187–192].

## Data Availability

Data sharing is not applicable to this article as no datasets were generated or analysed during the current study.

## Abbreviations

AUC: Area under the curve
CDS: Clinical decision support
CHALICE: Children’s Head Injury Algorithm for the Prediction of Important Clinical Events
CHARMS: Critical appraisal and data extraction for systematic reviews of prediction modelling studies
CT: Computed tomography
CTPA: Computed tomography pulmonary angiography
E.g.: For example
ED: Emergency department
GRADE: The Grading of Recommendations Assessment, Development and Evaluation
GRASP: Grading and assessment of predictive tools
I.e.: In other words or more precisely
ICU: Intensive care unit
IT: Information technology
LACE: Length of Stay, Admission Acuity, Comorbidity and Emergency Department Visits
MDCalc: Medical calculators
MEWS: Modified Early Warning Score
NASSS: Nonadoption, abandonment, and challenges to the scale-up, spread, and sustainability
PECARN: Paediatric Emergency Care Applied Research Network
RCTs: Randomised controlled trials
ROC: Receiver operating characteristic curve
TRIPOD: Transparent reporting of a multivariable prediction model for individual prognosis or diagnosis
UK: United Kingdom
US: United States
