# Supplementary material for: Developing a framework for evidence-based grading and assessment of predictive tools for clinical decision support
Source: BMC Med Inform Decis Mak. 2019 Oct 29;19:207. doi: 10.1186/s12911-019-0940-7 (PMC6820933; doi:10.1186/s12911-019-0940-7)

# Additional file 1

## Phases, Criteria, and Measures of Evaluation

Table S1. Phases, Criteria, and Measures of Evaluating Predictive Tools

| **Phase of Evaluation** | **Criteria of Evaluation** | **Definitions** | **Example Measures*** |
| --- | --- | --- | --- |
| Before Implementation | Predictive Performance | The ability of the predictive tool to utilise clinical variables and quantify relevant patient characteristics to produce an outcome that can be used to supports diagnostic, prognostic or therapeutic decisions made by clinicians and other healthcare professionals [[12](#_ENREF_12), [13](#_ENREF_13)]. | Discrimination:   - Sensitivity - Specificity - AUC, ROC, and C-Statistic - D-Statistic - Log-Rank Test.   Calibration:   - Calibration Plots & Curves - Hosmer-Lemeshow test - The Brier score. |
| During Implementation | Usability | The degree to which the predictive tool can be used by the specified users to achieve specified and quantifiable objectives in a specified context of use [[108](#_ENREF_108), [109](#_ENREF_109)]. | - Effectiveness of task management (accuracy and completeness). - Efficiency of utilising resources. - Users’ satisfaction, comfort with, and positive attitudes towards, the use of the tools. - Learnability - Memorability - Freedom of Errors. |
|  | Potential Effect | The expected, estimated or calculated impact of using the tool on different healthcare aspects, processes or outcomes, assuming the tool has been successfully implemented and used in the clinical practice, as designed by its developers [[41](#_ENREF_41), [101](#_ENREF_101)]. | - Clinical Effectiveness (Clinical Patient Outcomes). - Patient Safety (Complications, Side Effects, or Medical Errors). - Healthcare Efficiency (Utilisation of Resources, Such as Time and Money). |
| After Implementation | Post-Implementation Impact | The achieved change or influence of a predictive tool on different healthcare aspects, processes or outcomes, after the tool has been successfully implemented and used in the clinical practice, as designed by its developers [[2](#_ENREF_2), [42](#_ENREF_42)]. | - Clinical Effectiveness (Clinical Patient Outcomes). - Patient Safety (Complications, Side Effects, or Medical Errors). - Healthcare Efficiency (Utilisation of Resources, Such as Time and Money). |

* These measures of evaluation are examples, the list is not meant to be exhaustive; literature on predictive tools may evaluate them along other measures.

## Evaluating Evidence Direction

Table S2. Evaluating Evidence Direction Based on the Conclusions of Studies

| **Conclusions of Studies** | | | **Overall Direction of Evidence** |
| --- | --- | --- | --- |
| **Positive *** | **Equivocal **** | **Negative ***** |  |
| 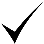 |  |  | Positive |
| 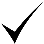 |  | 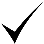 | Mixed |
| 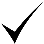 | 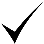 | 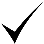 |  |
| 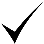 | 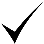 |  |  |
|  |  | 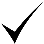 | Negative |
|  | 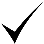 | 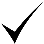 |  |
|  | 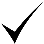 |  |  |
| * Positive Conclusion | - The tool shows positive valid predictive performance, usability, potential effect, or post-implementation impact, which are desirable and/or superior to other methods/tools, if the study includes a comparison. | | |
| ** Equivocal Conclusion | - The tool shows positive valid predictive performance or usability, which are acceptable, but not superior to other methods/tools, if the study includes a comparison. - The tool does not show positive potential effect or post-implementation impact. These are inferior to other methods/tools, if the study includes a comparison. | | |
| *** Negative Conclusion | - The tool shows that predictive performance or usability is poor, not acceptable, or inferior to other methods/tools, if the study includes a comparison. - The tool shows negative potential effect or post-implementation impact (leads to deterioration instead of improvement), whether in comparison or not. | | |

## GRASP Detailed Reports on Predictive Tools

Table S3. LACE Index for Readmission – Grade C1

| **Name** | LACE Index for Readmission | | | | | | | | | |
| --- | --- | --- | --- | --- | --- | --- | --- | --- | --- | --- |
| **Authors/Year** | Dr. Carl van Walraven, Canada, 2010 | | | | | | | | | |
| **Intended use** | Predicts 30 days readmission or death risk of medical and surgical inpatients after discharge | | | | | | | | | |
| **Intended user** | Used by nurses at patient discharge | | | | | | | | | |
| **Category** | Prognostic | | | | | | | | | |
| **Clinical area** | All medical/surgical areas | | | | | | | | | |
| **Target Population** | Hospitalised patients | | | | | | | | | |
| **Target Outcome** | 30 days readmission or death | | | | | | | | | |
| **Action** | Inform the clinical team about patients at high risk for readmission | | | | | | | | | |
| **Input source** | Objective data (Data is available in the EHR – electronic health record, or manually obtained from the patient medical record). | | | | | | | | | |
| **Input type** | Administrative data: Length of stay (days), Admission acuity (yes/no), Comorbidity (Charlson Index), Number of ED visits within 6 months. | | | | | | | | | |
| **Local context** | Input does not depend on local context of data | | | | | | | | | |
| **Methodology** | Multivariable logistic regression analysis | | | | | | | | | |
| **Endorsement** | Recommended by:   - Texas Healthcare Association, USA. - American Heart Association, USA. - Michigan Care Management Resource Center, USA | | | | | | | | | |
| **Automation Flag** | Manual | | | | | | | | | |
| **Tool Citations** | 455 | Reported in 7 studies | | | | | | | | |
| **Phase of Evaluation** | **Level of Evidence** | **Grade** | **Evaluation Studies** | | | | | | | |
| **Phase C:**  **Before implementation**  **Does the tool work? Is it possible?** | Internal validation | **C3** | Developed and tested for internal validity:   - van Walraven et al, 2010 [[125](#_ENREF_125)] | | | | | | | |
|  | External validation | **C2** | Tested for externally validity:   - Gruneir et al, 2011 [[130](#_ENREF_130)] | | | | | | | |
|  | External validation multiple times | **C1** | Tested for external validity again:   - Au et al, 2012 [[129](#_ENREF_129)]   Negative conclusion validation/performance studies:   - Cotter et al, 2012 [[131](#_ENREF_131)] - Wang et al, 2014 [[132](#_ENREF_132)] - Low et al, 2015 [[133](#_ENREF_133)] - Yu et al, 2015 [[134](#_ENREF_134)] | | | | | | | |
| **Phase B:**  **During implementation:**  **Is the tool practicable?** | Potential effect | **B2** | Not reported | | | | | | | |
|  | Usability | **B1** | Not reported | | | | | | | |
| **Phase A:**  **After implementation:**  **Is the tool desirable?** | Evaluation of Post-Implementation Impact on Clinical Effectiveness, Patient Safety or Healthcare Efficiency | **A3** | No subjective studies reported | | | | | | | |
|  |  | **A2** | No observational studies reported | | | | | | | |
|  |  | **A1** | No experimental studies reported | | | | | | | |
| **Final Grade** | **Grade C1** | | A1 | A2 | A3 | B1 | B2 | 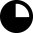 | C2 | 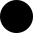 |
| **Direction of Evidence** | 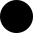 Positive Evidence | | 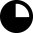 Mixed Evidence Supporting Positive Conclusion | | | | | | | |
|  | **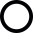** Negative Evidence | | 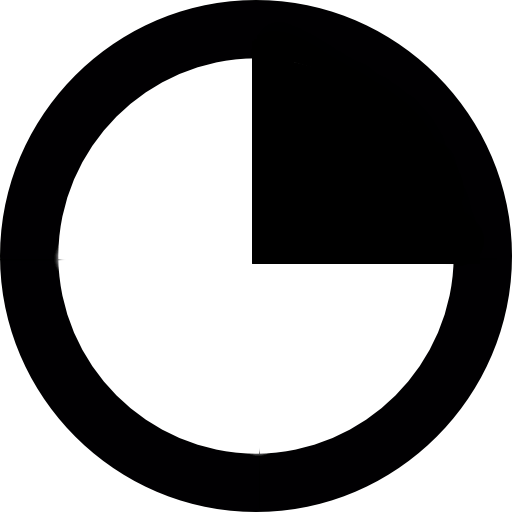 Mixed Evidence Supporting Negative Conclusion | | | | | | | |
| **Justification** | LACE Index is a prognostic tool designed to predict 30 days readmission or death after discharge from hospital. It uses multivariable logistic regression analysis of administrative data: length of stay, admission acuity, comorbidity (Charlson Comorbidity Index) and emergency department (ED) visits in the last six months, to produce a risk score [[125](#_ENREF_125)]. The tool has been tested for external validity twice: using a sample of 26,045 patients from six hospitals in Toronto and a sample of 59,652 patients from all hospitals in Alberta. The LACE Index showed external validity and superior predictive performance to previous tools endorsed by the Centres for Medicare and Medicaid Services [[129](#_ENREF_129), [130](#_ENREF_130)]. Two studies examined LACE Index predictive performance on small sub-population samples: 507 geriatric patients in the UK, and 253 congestive heart failure patients in the USA, and found that the index performed poorly [[131](#_ENREF_131), [132](#_ENREF_132)]. Two more studies reported that LACE Index works well but their own developed tools performed better [[133](#_ENREF_133), [134](#_ENREF_134)]. Using the mixed evidence protocol, the mixed evidence supports external validity, since the two negative conclusion studies have been conducted on very small samples of patients and a different subpopulation than the one LACE was developed for. There was no published evidence on the usability, potential effect or post-implementation impact of LACE Index. Accordingly, LACE Index has been assigned Grade C1. | | | | | | | | | |

Table S4. Centor Score for Streptococcal Pharyngitis – Grade B1

| **Name** | Centor Score for Streptococcal Pharyngitis | | | | | | | | | |
| --- | --- | --- | --- | --- | --- | --- | --- | --- | --- | --- |
| **Authors/Year** | Dr. Robert M. Centor, USA, 1981. Modified later by Dr. Warren McIsaac, Canada, 1998. | | | | | | | | | |
| **Intended use** | Estimate the probability that pharyngitis is streptococcal in adult patients presenting to the emergency department with sore throat | | | | | | | | | |
| **Intended user** | Used by physicians at ED as part of the clinical examination | | | | | | | | | |
| **Category** | Diagnostic | | | | | | | | | |
| **Clinical area** | Infectious diseases | | | | | | | | | |
| **Target Population** | Patients visiting the emergency department | | | | | | | | | |
| **Target Outcome** | Streptococcal pharyngitis | | | | | | | | | |
| **Action** | Consider rapid strep testing and/or culture | | | | | | | | | |
| **Input source** | Objective data (clinical examination) + Subjective data (symptoms described by patient) | | | | | | | | | |
| **Input type** | Clinical data: Age (3-14, 15-44 & >45 years), Exudate or swelling on tonsils (yes/no), Tender/swollen anterior cervical lymph nodes (yes/no), Temp >38°C (100.4°F) (yes/no), Cough (present/absent). Data is obtained from the patient. | | | | | | | | | |
| **Local context** | Input does not depend on local context of data | | | | | | | | | |
| **Methodology** | Rule-based algorithm | | | | | | | | | |
| **Endorsement** | Recommended by:   - Department of Health, New South Wales, Australia - American Academy of Family Physicians, United States - The National Institute for Health and Care Excellence, United Kingdom | | | | | | | | | |
| **Automation Flag** | Manual | | | | | | | | | |
| **Tool Citations** | 715 | Reported in 15 studies | | | | | | | | |
| **Phase of Evaluation** | **Level of Evidence** | **Grade** | **Evaluation Studies** | | | | | | | |
| **Phase C:**  **Before implementation**  **Does the tool work? Is it possible?** | Internal validation | **C3** | Developed and tested for internal validity:   - Centor et al, 1981 [[126](#_ENREF_126)] | | | | | | | |
|  | External validation | **C2** | Tested for external validity:   - Wigton, Connor & Centor, 1986 [[142](#_ENREF_142)] | | | | | | | |
|  | External validation multiple times | **C1** | Tested for external validity multiple times:   - Poses et al, 1986 [[141](#_ENREF_141)] - Meland, Digranes & Skjærven, 1993 [[140](#_ENREF_140)] - Ebell et al, 2000 [[137](#_ENREF_137)] - McIsaac et al, 2004 [[139](#_ENREF_139)] - Aalbers et al, 2011 [[135](#_ENREF_135)] - Fine, Nizet & Mandl, 2012 [[138](#_ENREF_138)] - Alper et al, 2013 [[136](#_ENREF_136)] | | | | | | | |
| **Phase B:**  **During implementation:**  **Is the tool practicable?** | Potential effect | **B2** | Not reported | | | | | | | |
|  | Usability | **B1** | Reported usability testing is positive:   - Feldstein et al, 2017 [[143](#_ENREF_143)] | | | | | | | |
| **Phase A:**  **After implementation:**  **Is the tool desirable?** | Evaluation of Post-Implementation Impact on Clinical Effectiveness, Patient Safety or Healthcare Efficiency | **A3** | No subjective studies reported | | | | | | | |
|  |  | **A2** | No observational studies reported | | | | | | | |
|  |  | **A1** | One RCT show positive post-implementation impact of Centor score on reducing unnecessary antibiotics prescribing:   - McIsaac et al, 1998 [[144](#_ENREF_144)]   One observational study + 3 RCTs show negative conclusions (No impact of Centor score on antibiotics prescribing):   - McIsaac et al, 1998 [[146](#_ENREF_146)] - Poses, Cebul & Wigton, 1995 [[147](#_ENREF_147)] - Worrall et al, 2007 [[148](#_ENREF_148)] - Little et al, 2014 [[145](#_ENREF_145)] | | | | | | | |
| **Final Grade** | **Grade B1** | | 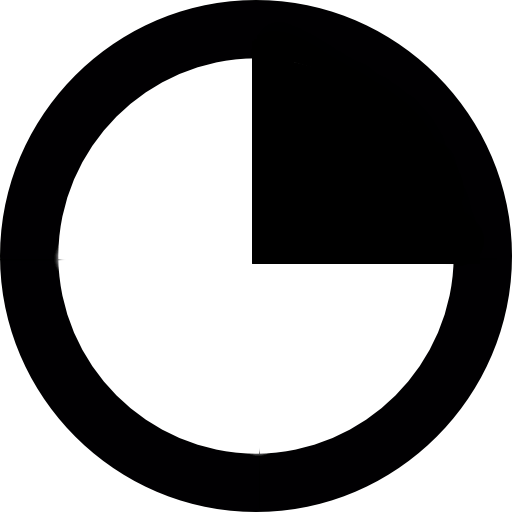 | A2 | A3 | 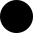 | B2 | 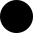 | C2 | 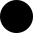 |
| **Direction of Evidence** | 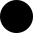 Positive Evidence | | 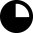 Mixed Evidence Supporting Positive Conclusion | | | | | | | |
|  | **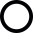** Negative Evidence | | 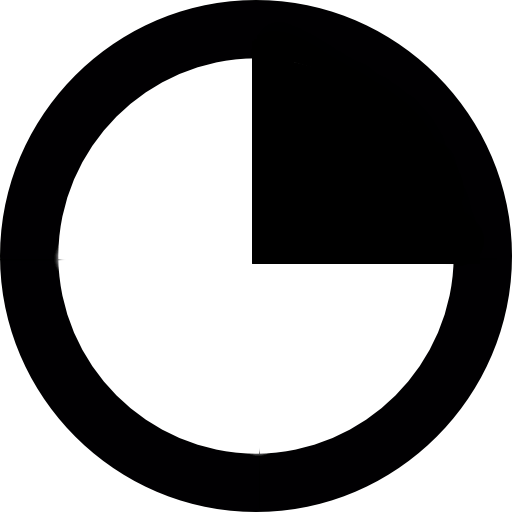 Mixed Evidence Supporting Negative Conclusion | | | | | | | |
| **Justification** | Centor score is a diagnostic tool that uses a rule-based algorithm on clinical data to estimate the probability that pharyngitis is streptococcal in adults who present to ED complaining of sore throat [[126](#_ENREF_126)]. The score has been tested for external validity multiple times and all studies reported positive conclusions [[135-142](#_ENREF_135)]. This qualifies Centor score for Grade C1. One study conducted a multicentre cluster RCT usability testing of the integration of Centor score into electronic health records. The study used “Think Aloud” testing with ten primary care providers, post interaction surveys in addition to screen captures and audio recordings to evaluate usability. Within the same study, another “Near Live” testing, with eight primary care providers, was conducted. Conclusions reported positive usability of the tool and positive feedback of users on the easiness of use and usefulness [[143](#_ENREF_143)]. This qualifies Centor score for Grade B1. Evidence of the post-implementation impact of Centor score post-implementation is mixed. One RCT conducted in Canada reported a clinically important 22% reduction in overall antibiotic prescribing [[144](#_ENREF_144)]. Four other studies, three of which were RCTs, reported that implementing Centor score did not reduce antibiotic prescribing in clinical practice [[145-148](#_ENREF_145)]. Using the mixed evidence protocol, the mixed evidence does not support positive post-implementation impact of Centor score. Therefore, Centor score has been assigned Grade B1. | | | | | | | | | |

Table S5. Wells’ Criteria for Pulmonary Embolism – Grade A2

| **Name** | Wells’ Criteria for Pulmonary Embolism | | | | | | | | | |
| --- | --- | --- | --- | --- | --- | --- | --- | --- | --- | --- |
| **Authors/Year** | Dr. Phil Wells, Canada, 1998. | | | | | | | | | |
| **Intended use** | Calculates the pre-test probability (risk)v of pulmonary embolism at the bedside without imaging | | | | | | | | | |
| **Intended user** | Used by physicians at ED as part of the clinical examination | | | | | | | | | |
| **Category** | Diagnostic | | | | | | | | | |
| **Clinical area** | Cardiovascular diseases | | | | | | | | | |
| **Target Population** | Patients visiting the emergency department | | | | | | | | | |
| **Target Outcome** | Pulmonary embolism | | | | | | | | | |
| **Action** | Rule out high risk patients with computed tomography angiography | | | | | | | | | |
| **Input source** | Objective data (clinical examination) + Subjective data (symptoms described by patient). | | | | | | | | | |
| **Input type** | Clinical data: Clinical signs and symptoms of DVT (yes/no), PE is #1 diagnosis OR equally likely (yes/no), Heart rate > 100 (yes/no), Immobilization at least 3 days OR surgery in the previous 4 weeks (yes/no), Previous, objectively diagnosed PE or DVT (yes/no), Haemoptysis (yes/no), Malignancy w/ treatment within 6 months or palliative (yes/no). | | | | | | | | | |
| **Local context** | Input does not depend on local context of data | | | | | | | | | |
| **Methodology** | Rule-based algorithm | | | | | | | | | |
| **Endorsement** | Recommended by:   - New South Wales Agency for Clinical Innovation, Australia - The Royal Australian College of General Practitioners, Australia | | | | | | | | | |
| **Automation Flag** | Manual | | | | | | | | | |
| **Tool Citations** | 1,260 | Reported in 13 studies | | | | | | | | |
| **Phase of Evaluation** | **Level of Evidence** | **Grade** | **Evaluation Studies** | | | | | | | |
| **Phase C:**  **Before implementation**  **Does the tool work? Is it possible?** | Internal validation | **C3** | Developed and tested for internal validity:   - Wells et al, 1998 [[124](#_ENREF_124)] - Wells et al, 2000 [[123](#_ENREF_123)] - Wells et al, 2001 [[127](#_ENREF_127)] | | | | | | | |
|  | External validation | **C2** | Tested for external validity:   - Page, 2006 [[151](#_ENREF_151)] | | | | | | | |
|  | External validation multiple times | **C1** | Tested for external validity multiple times:   - Gibson et al, 2008 [[150](#_ENREF_150)] - Klok et al, 2008 [[155](#_ENREF_155)] - Söderberg et al, 2009 [[153](#_ENREF_153)] - Geersing et al, 2012 [[149](#_ENREF_149)] - Arslan et al, 2013 [[154](#_ENREF_154)] - Posadas-Martínez et al, 2014 [[152](#_ENREF_152)] - Turan et al, 2017 [[156](#_ENREF_156)] | | | | | | | |
| **Phase B:**  **During implementation:**  **Is the tool practicable?** | Potential effect | **B2** | Not reported | | | | | | | |
|  | Usability | **B1** | Reported usability testing is positive:   - Press et al, 2015 [[157](#_ENREF_157)] | | | | | | | |
| **Phase A:**  **After implementation:**  **Is the tool desirable?** | Evaluation of Post-Implementation Impact on Clinical Effectiveness, Patient Safety or Healthcare Efficiency | **A3** | No subjective studies reported | | | | | | | |
|  |  | **A2** | Observational before-and-after intervention study showing positive post-implementation impact of Wells’ Criteria on healthcare efficiency:   - Murthy et al, 2016 [[158](#_ENREF_158)] | | | | | | | |
|  |  | **A1** | No experimental studies reported | | | | | | | |
| **Final Grade** | **Grade A2** | | A1 | 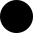 | A3 | 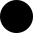 | B2 | 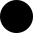 | C2 | 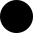 |
| **Direction of Evidence** | 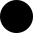 Positive Evidence | | 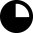 Mixed Evidence Supporting Positive Conclusion | | | | | | | |
|  | **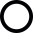** Negative Evidence | | 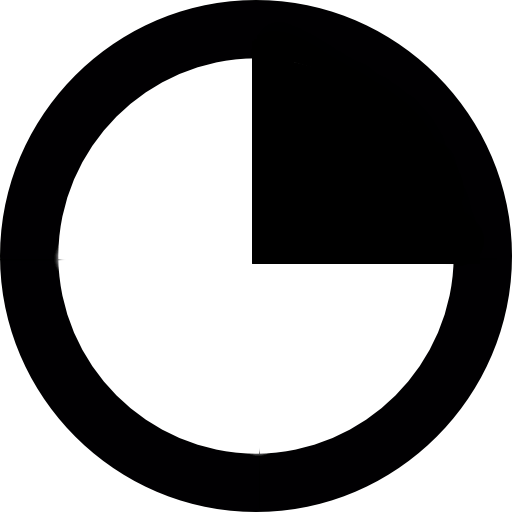 Mixed Evidence Supporting Negative Conclusion | | | | | | | |
| **Justification** | Wells’ criteria is a diagnostic tool used in ED to estimate pre-test probability of pulmonary embolism [[123](#_ENREF_123), [124](#_ENREF_124)]. Using a rule-based algorithm on clinical data, the tool calculates a score that excludes pulmonary embolism without diagnostic imaging [[127](#_ENREF_127)]. The tool was tested for external validity multiple times [[149-153](#_ENREF_149)] and its predictive performance has been also compared to other predictive tools [[154-156](#_ENREF_154)]. In all studies, Wells’ criteria was reported valid, which qualifies it for Grade C1. One study conducted usability testing for the integration of the tool into the electronic health record system of a tertiary care centre’s ED. The study identified a strong desire for the tool and received positive feedback on the usefulness of the tool itself. Subjects responded that they felt the tool was helpful, organized, and did not compromise clinical judgment [[157](#_ENREF_157)]. This qualifies Wells’ criteria for Grade B1. The post-implementation impact of Well’s Criteria on efficiency of computed tomography pulmonary angiography (CTPA) utilisation has been evaluated through an observational before-and-after intervention study. It was found that the Well’s Criteria significantly increased the efficiency of CTPA utilisation and decreased the proportion of inappropriate scans [[158](#_ENREF_158)]. Therefore, Well’s Criteria has been assigned Grade A2. | | | | | | | | | |

Table S6. Modified Early Warning Score (MEWS) – Grade A2

| **Name** | Modified Early Warning Score (MEWS) for Clinical Deterioration | | | | | | | | | |
| --- | --- | --- | --- | --- | --- | --- | --- | --- | --- | --- |
| **Authors/Year** | Dr. Christian Peter Subbe, UK, 2001 | | | | | | | | | |
| **Intended use** | Early detection of inpatients’ clinical deterioration, calculate chance of ICU admission or death within 60 days and potential need for higher levels of care. | | | | | | | | | |
| **Intended user** | Used by nurses at bedside | | | | | | | | | |
| **Category** | Prognostic | | | | | | | | | |
| **Clinical area** | General Medicine | | | | | | | | | |
| **Target Population** | Hospitalised patients | | | | | | | | | |
| **Target Outcome** | Clinical deterioration/death | | | | | | | | | |
| **Action** | Consider higher level of care for patient (e.g. transfer to ICU) | | | | | | | | | |
| **Input source** | Objective (Data from EHR – electronic health record) | | | | | | | | | |
| **Input type** | Clinical data: Systolic BP, Heart rate, Respiratory rate, Temperature, AVPU Score. | | | | | | | | | |
| **Local context** | Input does not depend on local context of data | | | | | | | | | |
| **Methodology** | Rule-based algorithm | | | | | | | | | |
| **Endorsement** | Recommended by:   - Australian Commission on Safety and Quality in Health Care, Australia - National Health Services, United Kingdom | | | | | | | | | |
| **Automation Flag** | Automated (However, in some hospitals a manual version is still used by nurses) | | | | | | | | | |
| **Tool Citations** | 1,176 | Reported in 13 studies | | | | | | | | |
| **Phase of Evaluation** | **Level of Evidence** | **Grade** | **Evaluation Studies** | | | | | | | |
| **Phase C:**  **Before implementation**  **Does the tool work? Is it possible?** | Internal validation | **C3** | Developed and tested for internal validity:   - Subbe et al, 2001 [[128](#_ENREF_128)] | | | | | | | |
|  | External validation | **C2** | Tested for External validity:   - Armagan et al, 2008 [[159](#_ENREF_159)] | | | | | | | |
|  | External validation multiple times | **C1** | Tested for external validity multiple times:   - Burch, Tarr & Morroni, 2008 [[160](#_ENREF_160)] - Dundar et al, 2016 [[161](#_ENREF_161)] - Gardner-Thorpe et al, 2006 [[162](#_ENREF_162)] - TANRIÖVER et al, 2016 [[164](#_ENREF_164)] - Wang et al, 2016 [[165](#_ENREF_165)] - Salottolo et al, 2017 [[163](#_ENREF_163)]   One negative conclusion validation/performance study:   - Tirotta et al, 2017 [[166](#_ENREF_166)] | | | | | | | |
| **Phase B:**  **During implementation:**  **Is the tool practicable?** | Potential effect | **B2** | Not reported | | | | | | | |
|  | Usability | **B1** | Not reported | | | | | | | |
| **Phase A:**  **After implementation:**  **Is the tool desirable?** | Evaluation of Post-Implementation Impact on Clinical Effectiveness, Patient Safety or Healthcare Efficiency | **A3** | No subjective studies reported | | | | | | | |
|  |  | **A2** | One observational before-and-after intervention study failed to prove positive post-implementation impact of the MEWS on patient safety:   - Subbe et al, 2003 [[167](#_ENREF_167)]   Three observational before-and-after intervention studies showed positive post-implementation impact of the MEWS on patient safety:   - Moon et al, 2011 [[170](#_ENREF_170)] - De Meester et al, 2013 [[168](#_ENREF_168)] - Hammond et al, 2013 [[169](#_ENREF_169)] | | | | | | | |
|  |  | **A1** | No experimental studies reported | | | | | | | |
| **Tool Grade** | **Grade A2** | | A1 | 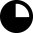 | A3 | B1 | B2 | 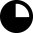 | C2 | 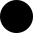 |
| **Direction of Evidence** | 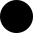 Positive Evidence | | 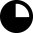 Mixed Evidence Supporting Positive Conclusion | | | | | | | |
|  | **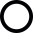** Negative Evidence | | 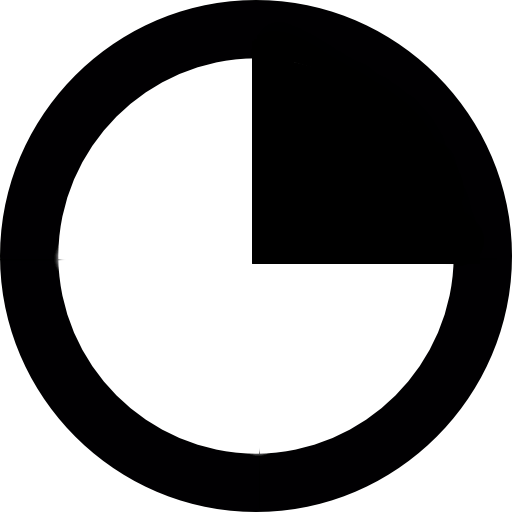 Mixed Evidence Supporting Negative Conclusion | | | | | | | |
| **Justification** | The MEWS is a prognostic tool for early detection of inpatients’ clinical deterioration and potential need for higher levels of care. The tool uses a rule-based algorithm on clinical data to calculate a risk score [[128](#_ENREF_128)]. The MEWS has been tested for external validity multiple times in different clinical areas, settings and populations [[159-165](#_ENREF_159)]. All studies reported the tool is externally valid. However, one study reported MEWS poorly predicted the in-hospital mortality risk of patients with sepsis [[166](#_ENREF_166)]. Using the mixed evidence protocol, the mixed evidence supports external validity, qualifying MEWS for Grade C1. No literature has been found regarding its usability or potential effect. The MEWS has been implemented in different healthcare settings. One observational before-and-after intervention study failed to prove positive post-implementation impact of the MEWS on patient safety in acute medical admissions [[167](#_ENREF_167)]. However, three more recent observational before-and-after intervention studies reported positive post-implementation impact of the MEWS on patient safety. One study reported significant increase in frequency of patient observation and decrease in serious adverse events after intensive care unit (ICU) discharge [[168](#_ENREF_168)]. The second reported significant increase in frequency of vital signs recording, 24h post-ICU discharge and 24h preceding unplanned ICU admission [[169](#_ENREF_169)]. The third, an eight years study, reported that the post-implementation four years showed significant reductions in the incidence of cardiac arrests, the proportion of patients admitted to ICU and their in-hospital mortality [[170](#_ENREF_170)]. Using the mixed evidence protocol, the mixed evidence supports positive post-implementation impact. The MEWS has been assigned Grade A2. | | | | | | | | | |

Table S7. Ottawa Knee Rule – Grade A1

| **Name** | Ottawa Knee Rule | | | | | | | | | |
| --- | --- | --- | --- | --- | --- | --- | --- | --- | --- | --- |
| **Authors/Year** | Dr. Ian Stiell, Canada, 1995 | | | | | | | | | |
| **Intended use** | Exclude the need for an X-ray for possible bone fracture in adult patients | | | | | | | | | |
| **Intended user** | Used by emergency physicians as part of the clinical examination | | | | | | | | | |
| **Category** | Diagnostic | | | | | | | | | |
| **Clinical area** | Orthopaedics | | | | | | | | | |
| **Target Population** | Patients visiting the emergency department | | | | | | | | | |
| **Target Outcome** | Bone fracture | | | | | | | | | |
| **Action** | Refer patient to knee imaging | | | | | | | | | |
| **Input source** | Objective data (clinical examination) + Subjective data (symptoms described by patient) | | | | | | | | | |
| **Input type** | Clinical data: Age ≥55 (yes/no), Isolated tenderness of the patella (no other bony tenderness) (yes/no), Tenderness at the fibular head (yes/no), Unable to flex knee to 90° (yes/no), Unable to bear weight both immediately and in ED (4 steps, limping is okay) (yes/no). Data is obtained from the patient. | | | | | | | | | |
| **Local context** | Input does not depend on local context of data | | | | | | | | | |
| **Methodology** | Set of rules | | | | | | | | | |
| **Endorsement** | Recommended by:   - Department of Emergency Medicine, Faculty of medicine, Ottawa University, Canada‎ - The Royal College of Radiologists, United Kingdom - The National Institute for Health and Care Excellence, United Kingdom | | | | | | | | | |
| **Automation Flag** | Manual | | | | | | | | | |
| **Tool Citations** | 227 | Reported in 15 studies | | | | | | | | |
| **Phase of Evaluation** | **Level of Evidence** | **Grade** | **Evaluation Studies** | | | | | | | |
| **Phase C:**  **Before implementation**  **Does the tool work? Is it possible?** | Internal validation | **C3** | Developed and tested for internal validity:   - Stiell et al, 1995 [[122](#_ENREF_122)] | | | | | | | |
|  | External validation | **C2** | Tested for externally validity | | | | | | | |
|  | External validation multiple times | **C1** | Externally tested for externally validity (One systematic review reported 11 validation studies):   - Bachmann et al, 2004 [[171](#_ENREF_171)] | | | | | | | |
| **Phase B:**  **During implementation:**  **Is the tool practicable?** | Potential effect | **B2** | Not reported | | | | | | | |
|  | Usability | **B1** | Not reported | | | | | | | |
| **Phase A:**  **After implementation:**  **Is the tool desirable?** | Evaluation of Post-Implementation Impact on Clinical Effectiveness, Patient Safety or Healthcare Efficiency | **A3** | No subjective studies reported | | | | | | | |
|  |  | **A2** | No observational studies reported | | | | | | | |
|  |  | **A1** | Two nonrandomised controlled studies reported positive post-implementation impact of Ottawa knee rule on healthcare efficiency:   - Stiell et al, 1997 [[172](#_ENREF_172)] - Nichol et al, 1999 [[173](#_ENREF_173)] | | | | | | | |
| **Final Grade** | **Grade A1** | | 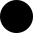 | A2 | A3 | B1 | B2 | 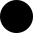 | C2 | 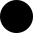 |
| **Direction of Evidence** | 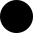 Positive Evidence | | 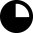 Mixed Evidence Supporting Positive Conclusion | | | | | | | |
|  | **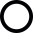** Negative Evidence | | 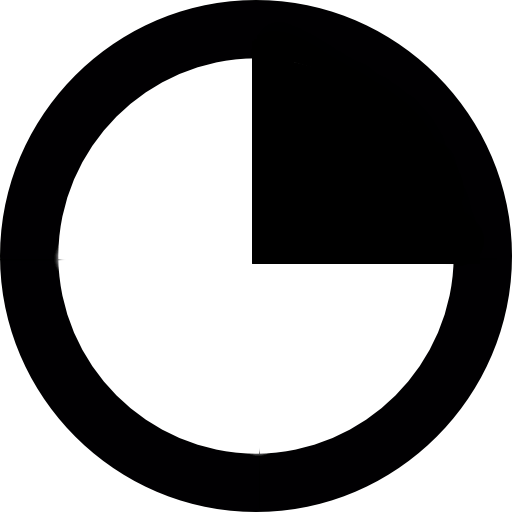 Mixed Evidence Supporting Negative Conclusion | | | | | | | |
| **Justification** | Ottawa knee rule is a diagnostic tool used to exclude the need for an X-ray for possible bone fracture in patients presenting to the ED, using a simple five items manual check list [[122](#_ENREF_122)]. It is one of the oldest, most accepted and successfully used rules in CDS. The tool has been tested for external validity multiple times. One systematic review identified 11 studies, 6 of them involved 4,249 adult patients and were appropriate for pooled analysis, showing high sensitivity and specificity [[171](#_ENREF_171)]. Furthermore, two studies discussed the impact of implementing Ottawa knee rule on healthcare efficiency. One nonrandomised controlled trial with before-after and concurrent controls included a total of 3,907 patients seen during two 12-month periods before and after the intervention. The study reported that the rule decreased the use of knee radiography without patient dissatisfaction or missed fractures and was associated with reduced waiting times and costs per patient [[172](#_ENREF_172)]. Another nonrandomised controlled trial reported that the proportion of ED patients referred for knee radiography was reduced. The study also reported that the practice based on the rule was associated with significant cost savings [[173](#_ENREF_173)]. The Ottawa knee rule has been assigned Grade A1. | | | | | | | | | |

## Predictive Performance, Usability and Post-implementation Impact Tables of Predictive Tools

Table S8: Predictive Performance of the Five Tools – Before Implementation

| **Tool** | **Discrimination** | | **Calibration** |
| --- | --- | --- | --- |
|  | **AUC/C-Statistic** | **Sensitivity, Specificity, Cut-Off** | **Hosmer–Lemeshow**  **goodness-of-fit** |
| LACE Index | - 0.68 (95% CI, 0.68–0.69) [[125](#_ENREF_125)] - 0.68 [[129](#_ENREF_129)] - 0.56 (95% CI, 0.46–0.66) [[187](#_ENREF_187)] | - 66.3%, 53.3%, 50% [[133](#_ENREF_133)] | - 14.1 (P=0.59) [[125](#_ENREF_125)] |
| Centor Score | - 0.78 [[126](#_ENREF_126)] - 0.72 [[138](#_ENREF_138)] - 0.84 [[136](#_ENREF_136)] | - 90%, 92%, 50% [[126](#_ENREF_126)] - 49%, 82%, 50% [[135](#_ENREF_135)] - 92%, 73%, 50% [[139](#_ENREF_139)] - 92%, 63%, 50% [[136](#_ENREF_136)] | - Not reported |
| Wells' Criteria | - 0.71 [[153](#_ENREF_153)] - 0.75 [[154](#_ENREF_154)] - 0.79 (95% CI, 0.75-0.82) [[152](#_ENREF_152)] - 0.79 (95% CI, 0.72–0.87) [[155](#_ENREF_155)] - 0.76 [[156](#_ENREF_156)] - 0.74 (95% CI,0.72-0.76) [[150](#_ENREF_150)] | - 83%, 48%, 50% [[153](#_ENREF_153)] - 65%, 81%, 50% [[152](#_ENREF_152)] - 100%, 56%, 50% [[156](#_ENREF_156)] - 95%, 51%, 50% [[149](#_ENREF_149)] | - Not reported |
| MEWS | - 0.73 (95% CI, 0.69–0.77) –Hospitalisation [[161](#_ENREF_161)] - 0.89 (95% CI 0.84–0.94) – In-hospital mortality [[161](#_ENREF_161)] - 0.79 (95% CI, 0.74-0.83) – Mortality [[163](#_ENREF_163)] - 0.56 (95% CI, 0.51 to 0.62) – ICU Admission [[163](#_ENREF_163)] - 0.85 (95% CI, 0.77–0.91) [[164](#_ENREF_164)] - 0.80 (95% CI, 0.72–0.88) [[37](#_ENREF_37)] - 0.76 – Mortality [[188](#_ENREF_188)] | - 88%, 68%, 50% (MEWS≥3) [[162](#_ENREF_162)] - 53%, 91%, 50% (MEWS≥4) Mortality [[163](#_ENREF_163)] - 17%, 94%, 50% (MEWS≥4) ICU Admission [[163](#_ENREF_163)] - 86%, 94%, 50% (MEWS≥4) [[164](#_ENREF_164)] - 75%, 83%, 50% (MEWS≥4) [[189](#_ENREF_189)] - 57%, 86%, 50% (MEWS≥4) [[188](#_ENREF_188)] | - P=0.06 [[190](#_ENREF_190)] |
| Ottawa Knee Rule | - Not reported | - 98.5%, 48.6%, 50% [[171](#_ENREF_171)]* - 100% [[172](#_ENREF_172)] - 100%, 42.8%, 50% [[191](#_ENREF_191)] - 95%, 44%, 50% [[192](#_ENREF_192)] | - Not reported |

* A systematic review study.

Table S9. Usability of Two Predictive Tools – During Implementation

| **Tool** | **Study Type** | **Method** | **Outcomes** |
| --- | --- | --- | --- |
| Centor Score | Usability testing [[143](#_ENREF_143)] | Think Aloud + Near Live | - Positive usability & feedback of users   - Easiness of use   - Usefulness |
| Wells' Criteria | Usability testing [[157](#_ENREF_157)] | Think Aloud + Near Live | - Positive usability & feedback of users   - Tool is helpful   - Organized   - Did not compromise clinical judgment |

Table S10. Post-Implementation Impact of Three Predictive Tools

| **Tool** | **Study Type** | **Study Settings** | **Outcome** | **Effect Size** |
| --- | --- | --- | --- | --- |
| Wells' Criteria | Prospective before-and-after intervention study [[158](#_ENREF_158)] | Public-sector tertiary-level and referral teaching hospital in South Africa | Efficiency of CTPA utilisation | 17.4% vs 30.7% (p=0.036) |
|  |  |  | Inappropriate CTPA scans | 82.6% vs 69.3% (p=0.015) |
| MEWS | Prospective before-and-after intervention study [[168](#_ENREF_168)] | A University Hospital, in Belgium | Frequency of patient observation | 0.99 vs 1.07 (p=0.005) |
|  |  |  | Serious adverse events after ICU discharge | 5.7% vs 3.5% |
|  | Prospective before-and-after intervention study [[169](#_ENREF_169)] | The department of intensive care medicine, at a tertiary referral hospital in Brisbane,  Australia | Vital signs documentation after ICU discharge | 210% (95% CI 148, 288%, p <0.001). |
|  |  |  | Vital signs documentation before unplanned ICU admissions | 44% (95% CI, 3, 102%, p = 0.035). |
|  | Retrospective analysis of prospectively collected data before-and-after intervention study [[170](#_ENREF_170)] | The department of perioperative and critical care at a university teaching hospital in the United Kingdom | Cardiac arrest calls | 0.2% vs 0.4% (p<0.0001) |
|  |  |  | Patients admitted to ICU | 2% vs 3% (p=0.004) |
|  |  |  | In-hospital mortality of cardiac arrest patients | 42% vs 52% (p=0.05) |
| Ottawa Knee Rule | Nonrandomised controlled trial with before-after & concurrent controls [[172](#_ENREF_172)] | The Emergency departments of two teaching and two community hospitals in Canada | Reduced time spent by patient | 85.7 minutes vs 118.8 minutes |
|  |  |  | Cost savings per patient | US $80 vs US $183 |
|  | Nonrandomised controlled trial with before-after & concurrent controls [[173](#_ENREF_173)] | The Emergency departments of an academic and a community hospital in Canada. | Reduced proportion of knee injury patients referred to radiology | 77.6% vs 57.1% |
|  |  |  | Cost savings per patient | $31 (95% CI, 22 to 44) to $34 (95% CI, 24 to 47). |

## Study Selection Process

**Figure S1.** Study Selection for the Focused Review of Literature

## Searching the Literature for Published Evidence on Predictive Tools

**Figure S2.** Searching the Literature for Published Evidence on Predictive Tools

## The Mixed Evidence Protocol

**Figure S3.** The Mixed Evidence Protocol

The mixed evidence protocol is based on four steps. Firstly, it considers the degree of matching between the evaluation study conditions and the original tool specifications, in terms of the predictive task, outcome, intended use and users, clinical specialty, healthcare settings, target population, and age group. Secondly, it considers the quality of the study, in terms of sample size, data collection, study methods, and credibility of institute or authors. Based on these two criteria, the studies in the mixed evidence on the tool are classified into 1) Class A: matching evidence of high quality, 2) Class B: matching evidence of low quality or non-matching evidence of high quality, and 3) Class C: non-matching evidence of low quality. Thirdly, it considers the evidence conclusion on the reported evaluation criteria; the predictive performance, potential effect, usability, and post-implementation impact. In the fourth step, studies evaluating predictive tools in closely matching conditions to the tool specifications and providing high quality evidence, Class A, are considered first; taking into account their conclusions on the evaluation criteria in deciding the overall direction of evidence. On the other hand, studies evaluating predictive tools in different conditions to the tool specifications and providing low quality evidence, Class C, are considered last. The conclusion of one study in Class A is considered a stronger evidence than the conflicting conclusions of any number of studies in Class B or C, and the overall direction of the evidence is decided towards the conclusion of the study of Class A. When multiple studies of the same class; for example Class A, report conflicting conclusions, then we compare the number of studies reporting positive conclusions to those reporting negative conclusions and the overall direction of the evidence is decided towards the conclusions of the larger group. If the two groups are of the same size, then we check if there are more studies in other classes, if not then we examine the reported evaluation criteria and their values in the two groups of studies.

## Performance Figures of Predictive Tools

**Figure S4.** Reported C-Statistic of LACE Index, Centor Score, Wells Criteria and MEWS


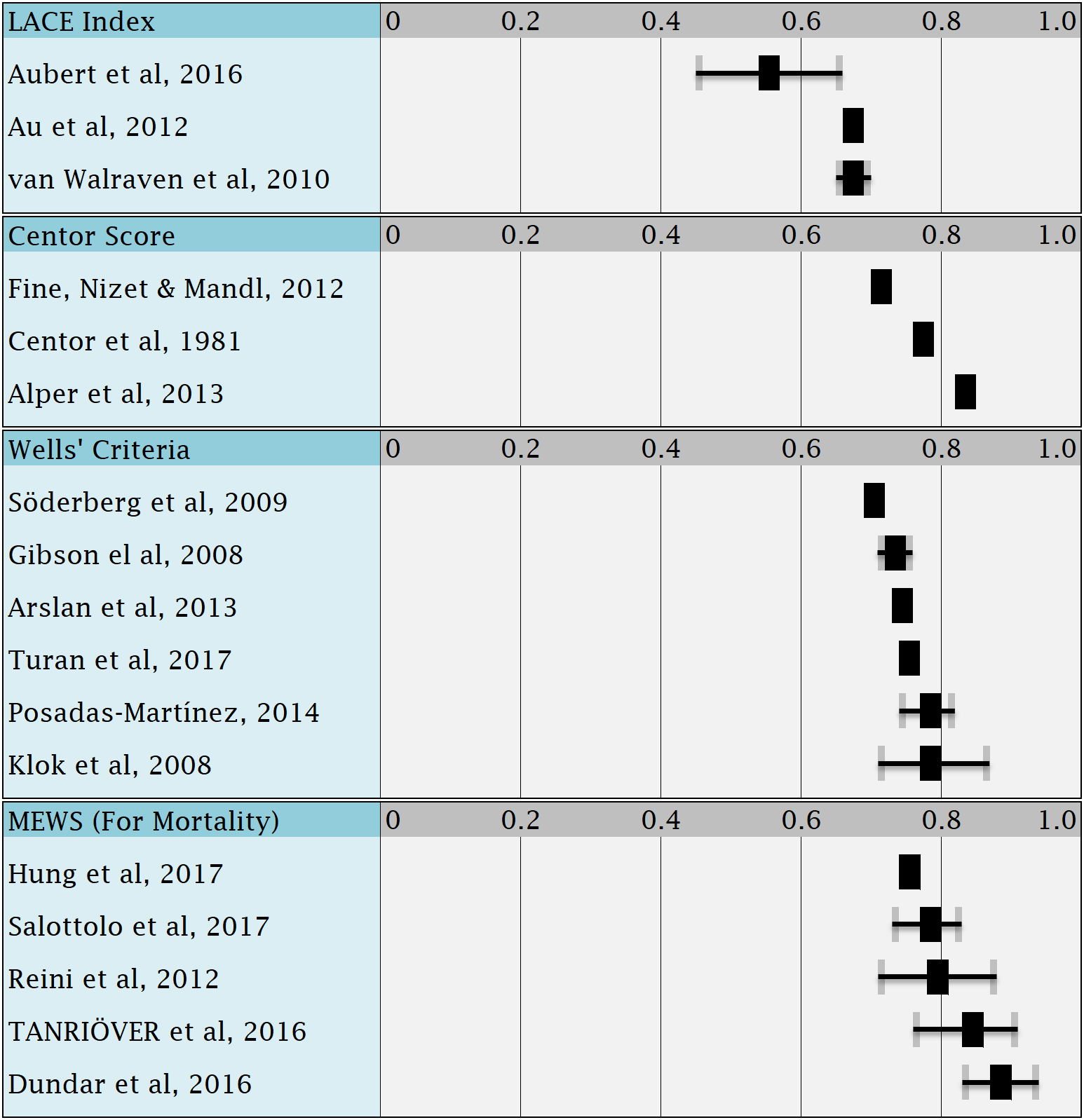

Supplement: Supplementary file 1 — Additional file 1: Table S1. Phases, Criteria, and Measures of Evaluating Predictive Tools. Table S2. Evaluating Evidence Direction Based on the Conclusions of Studies. Table S3. LACE Index for Readmission – Grade C1. Table S4. Centor Score for Streptococcal Pharyngitis – Grade B1. Table S5. Wells’ Criteria for Pulmonary Embolism – Grade A2. Table S6. Modified Early Warning Score (MEWS) – Grade A2. Table S7. Ottawa Knee Rule – Grade A1. Table S8. Predictive Performance of the Five Tools – Before Implementation. Table S9. Usability of Two Predictive Tools – During Implementation. Table S10. Post-Implementation Impact of Three Predictive Tools. Figure S1. Study Selection for the Focused Review of Literature. Figure S2. Searching the Literature for Published Evidence on Predictive Tools. Figure S3. The Mixed Evidence Protocol. Figure S4. Reported C-Statistic of LACE Index, Centor Score, Wells Criteria and MEWS [187–192]. [file 12911_2019_940_MOESM1_ESM.docx]
